# Binding and entering: COVID finds a new home

**DOI:** 10.1371/journal.ppat.1009857

**Published:** 2021-08-30

**Authors:** Michelle N. Vu, Vineet D. Menachery

**Affiliations:** 1 Department of Microbiology and Immunology, University of Texas Medical Branch, Galveston, Texas, United States of America; 2 Institute for Human Infections and Immunity, University of Texas Medical Branch, Galveston, Texas, United States of America; Mount Sinai School of Medicine, UNITED STATES

## Abstract

Severe Acute Respiratory Syndrome Coronavirus 2 (SARS-CoV-2) emerged as a virus with a pathogenicity closer to Severe Acute Respiratory Syndrome Coronavirus (SARS-CoV) and Middle East Respiratory Syndrome Coronavirus (MERS-CoV) and a transmissibility similar to common cold coronaviruses (CoVs). In this review, we briefly discuss the features of the receptor-binding domain (RBD) and protease cleavage of the SARS-CoV-2 spike protein that enable SARS-CoV-2 to be a pandemic virus.

## Introduction

A novel coronavirus (CoV), subsequently named Severe Acute Respiratory Syndrome Coronavirus 2 (SARS-CoV-2), emerged in December 2019 and has caused approximately 177 million cases and approximately 3.8 million deaths worldwide (global case fatality rate (CFR) of 2.2% varying in individual countries) [1]. SARS-CoV-2 is not the first CoV to have caused a pandemic, as both Severe Acute Respiratory Syndrome Coronavirus (SARS-CoV) and Middle East Respiratory Syndrome Coronavirus (MERS-CoV) have done so previously in 2002 and 2012, respectively [2]. While the CFRs of SARS-CoV and MERS-CoV are greater than SARS-CoV-2 (11% and 34%, respectively), the earlier endemics did not reach the same scale (8,400 and approximately 2,500 cases, respectively). In contrast, human coronaviruses (hCoV-229E, hCoV-NL63, hCoV-OC43, and hCoV-HKU1) cause limited, common cold symptoms, yet have been endemic globally for decades [3]. Upon examination, there appears to be an inverse relationship between virus transmissibility and pathogenicity—the less pathogenic hCoVs have robust transmission, the highly pathogenic SARS-CoV and MERS-CoV have limited transmission, and the current SARS-CoV-2 appears to have established a rare balance between the two.

A similar trend between transmissibility and pathogenicity can be seen with influenza viruses. Like hCoVs, seasonal influenza viruses are common globally, characterized by high transmissibility, low pathogenicity with approximately 20% of the population infected, and a CFR less than 1% annually [4]. Analogous to SARS-CoV and MERS-CoV, high pathogenicity influenza strains such as H5N1 and H7N9 influenza viruses have high CFR (53% and 39%, respectively), but low transmission with approximately 1,550 and approximately 850 cases since their introduction [5,6]. The 1918 H1N1 influenza virus is comparable to SARS-CoV-2 as it achieved a balance between transmissibility and pathogenicity, with 1/3 of the world’s population infected and approximately 50 million deaths (CFR ≈ 2.5%) [7]. More recently, the H1N1 subtype caused another pandemic in 2009 with relatively robust transmissibility, although with lower pathogenicity (CFR <0.02%) [8].

**Fig 1:**
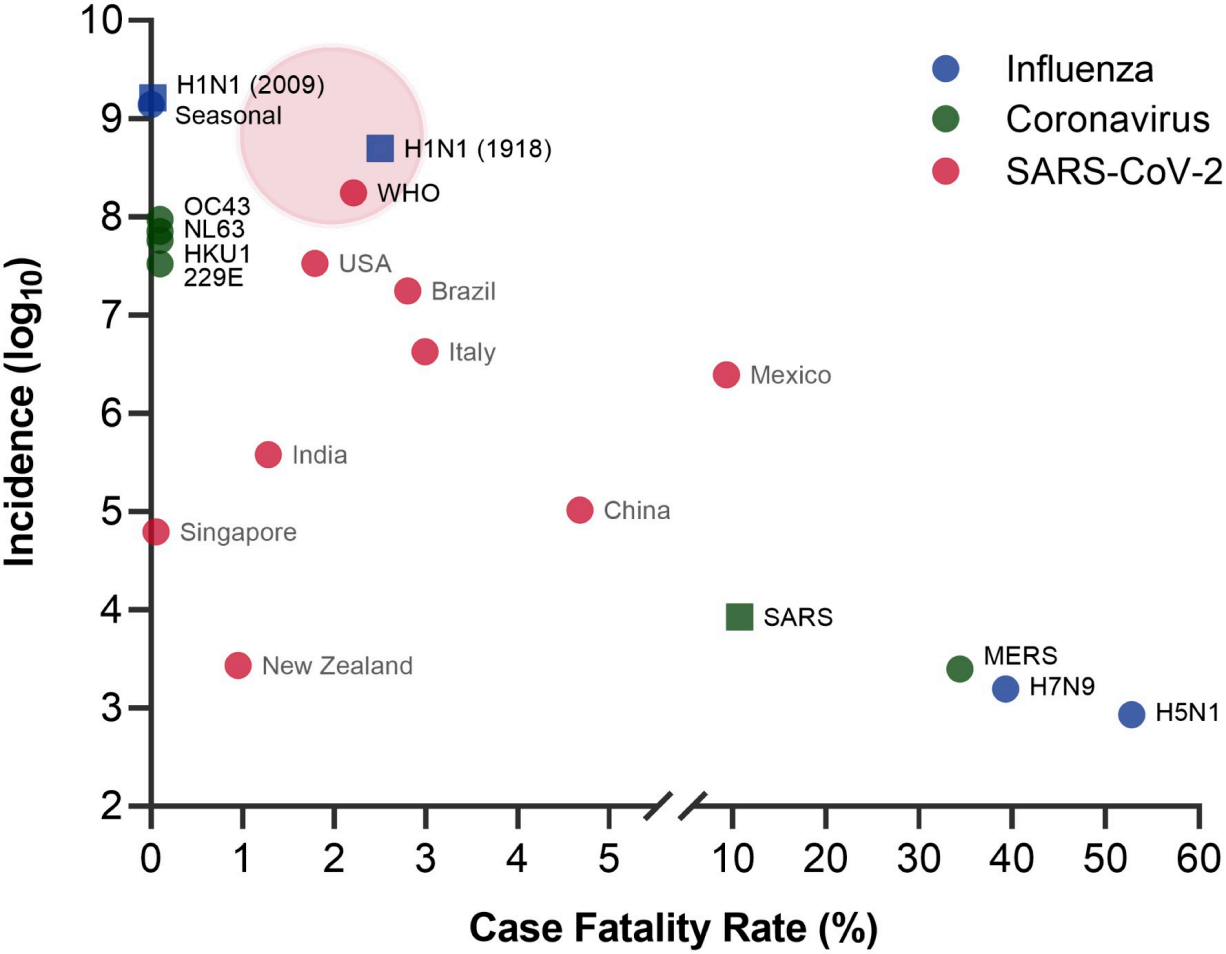
Transmission and pathogenesis of CoVs and influenza viruses. An inverse relationship exists between transmission (incidence) and pathogenesis (CFR) as the more transmissible viruses are less pathogenic (e.g., hCoVs and seasonal influenza) and vice versa (e.g., MERS and H5N1 influenza). SARS-CoV-2 has established a balance displaying high transmissibility and moderate pathogenesis, similar to the 1918 H1N1 influenza. The CFR of SARS-CoV-2 vary among individual countries, with WHO representing the global values. The red cloud represents an estimated range of the final SARS-CoV-2 incidence and CFR. ● represents ongoing or seasonal values; ■ represents historical values. CFR, case fatality rate; CoV, coronavirus; hCoV, human coronavirus; MERS, Middle East Respiratory Syndrome; SARS, Severe Acute Respiratory Syndrome; SARS-CoV-2, Severe Acute Respiratory Syndrome Coronavirus 2; WHO, World Health Organization.

The idea of a trade-off between virulence and transmission of pathogens has long been proposed with few empirical evidence to attest to it [9,10]. However, such a relationship is clear when studying CoVs and influenza viruses (Fig 1). Both groups of viruses have strains capable of great harm (SARS-CoV, MERS-CoV, H5N1, and H7N9) yet are limited in transmission. In contrast, strains that cause mild disease (hCoVs and seasonal influenza) have been endemic globally. Then, there are viruses (SARS-CoV-2 and 1918 H1N1) that have established a key balance, capable of achieving devastating pathogenicity, but minimal loss in transmissibility. While only a few viruses have attained this balance, it is imperative to understand how these viruses emerged in order to head off the next pandemic. In this review, we focus on the start of infection, examining the impact of receptor binding and protease activation on virus tropism, transmissibility, and pathogenicity.

### Location, location, location

The location in which the virus replicates along the respiratory tract guides how the virus is able to transmit and its pathogenicity. Traditionally, viruses that bind in the upper airways exhibit lower pathogenicity compensated with high transmissibility; the inverse is true for those that bind lower in the airways. For example, CoVs associated with the common cold (hCoV-229E, hCoV-NL63, hCoV-OC43, and hCoV-HKU1) replicate primarily in the upper respiratory tract including the nasal and oropharyngeal areas [11]. Testing for these less virulent hCoVs typically finds positives from upper airways samples and rarely finds lower airway infection. In turn, upper airway replication may correspond with greater transmissibility. In contrast, both SARS-CoV and MERS-CoV rarely induced positive tests from upper airway samples [12,13]. Instead, during the SARS-CoV outbreak and the later MERS-CoV emergence, samples from deep in the respiratory tract (bronchiolar lavage and sputum) were critical for diagnosis. These results suggest that the more virulent CoVs induced more replication and damage deep in the lung. The corresponding loss in lung function directly plays a role in the increased lethality associated with each. It also may explain relatively low transmission outside hospital settings and super-spreaders.

For SARS-CoV-2, a balance exists with the infection of both the upper and lower airways. Early on, testing of nasopharyngeal swabs confirmed upper airway infection with SARS-CoV-2; subsequent work found positive samples in saliva and nasal swabs suggesting replication throughout the upper airways [14]. However, lung infection as evidenced by bronchoalveolar lavage (BAL), sputum, and severe disease demonstrate robust replication in the lung in a large subset of patients [14]. This even distribution in the upper and lower airways highlight and correspond to the transmissibility and pathogenicity relationship highlighted above (Fig 1) and may offer a primer for why SARS-CoV-2 and Coronavirus Disease 2019 (COVID-19) has had such a broad impact on society.

### Finding the key

Location of virus replication is in part due to receptor recognition and binding. For influenza, the viruses’ interactions between hemagglutinin and sialylated glycan receptors have been well characterized [15]. Briefly, sialic acid is linked to galactose through either an α2,3 (Saα2,3) or α2,6 (Saα2,6) linkage, which are expressed in an inverse gradient along the human respiratory tract—Saα2,6 more highly expressed in the upper airways and Saα2,3 in the lower airways [15,16]. The hemagglutinin of high pathogenicity avian influenza viruses (e.g., H7N9 and H5N1) preferentially bind to SAα2,3, directing their replication to the lower airways, limiting their transmission, but inducing more severe disease [16–18]. Conversely, human influenza viruses (e.g., H1N1 and H3N2) typically do not cause significant pathogenicity and have been observed to bind more to Saα2,6 in the upper airways, therefore affording them greater transmission opportunities [16,18]. Indeed, influenza viruses that are more transmissible via air preferentially replicate in the upper airways [19].

CoVs bind to their receptors through their spike protein, with different CoV spikes being similar at their core, but different enough in their globular head to utilize a variety of receptors. Most CoVs bind to membrane ectopeptidases—hCoV-229E to aminopeptidase N (APN); MERS-CoV to dipeptidyl peptidase-4 (DPP4); and hCoV-NL63, SARS-CoV, and SARS-CoV-2 to angiotensin converting enzyme 2 (ACE2) [20,21]. The receptors for hCoV-OC43 and hCoV-HKU1 are unknown, although they interact with acetylated sialic acids [22]. Interestingly, hCoV-NL63, SARS-CoV, and SARS-CoV-2 share the same receptor yet display distinct infection and virulence. A key to this discrepancy may be the receptor binding affinity of their spike protein. Compared to the SARS-CoV spike protein, hCoV-NL63 spike protein has less affinity to human angiotensin converting enzyme 2 (hACE2), while SARS-CoV-2 spike protein has comparable or slightly greater affinity [23,24]. ACE2 expression follows a gradient along the respiratory tract, with greater expression in the upper airways and decreasing further down [25]. One hypothesis argues that the strength receptor affinity may correlate with receptor abundance in an inverse relationship. On one end of the spectrum, the lower receptor affinity of hCoVs may hamper potential lower respiratory infection where ACE2 is less abundant, resulting in replication primarily in the upper airways where the greater ACE2 expression offers more opportunities for binding. On the opposite end, SARS-CoV replication is predominantly observed in the lower airways, perhaps due to its strong receptor affinity allowing binding to fewer ACE2 when swept deeper. Interestingly, although the SARS-CoV-2 spike protein has similar receptor affinity as to the SARS-CoV, its infection is observed throughout the respiratory tract and with more replication in the upper airways [25]. Receptor affinity is therefore not solely responsible for where the virus replicates and subsequent effects on transmission and virulence. The spike protein interacts with other host proteins, specifically proteases, to establish successful entry and infection.

### Cutting the lock

While a virus may be able to bind to its receptor, other factors are needed to establish replication. Upon receptor binding, both CoVs and influenza viruses undergo an uncoating and fusion process to release the viral genomes. Both the CoV spike protein and the influenza virus hemagglutinin are type I fusion proteins; following receptor binding, the proteins are cleaved by host proteases to prime fusion and entry with the host cell. Host proteases, such as trypsin, transmembrane serine protease 2 (TMPRSS2), furin and cathepsin, are ubiquitously expressed throughout the respiratory tract. However, due to variations in the cleavage sequences, not all proteases are the compatible. As such, the proteases used by a particular virus for cell entry may be key to its tropism.

Revisiting the previous comparison, hCoV-NL63, SARS-CoV, and SARS-CoV-2 use different host proteases despite sharing the hACE2 receptor. While hCoV-NL63 spike protein cleavage has not been studied in depth, TMPRSS2 is involved in its infection as well as in SARS-CoV and SARS-CoV-2 infection [26,27]. SARS-CoV is also capable of utilizing cathepsin while hCoV-NL63 cannot, which may explain differences in tropism [28]. Interestingly, SARS-CoV-2 spike protein has a polybasic furin cleavage site, PRRA, directly upstream of the S1/S2 cleavage site not found in other group 2B CoVs; however, furin cleavage sites are found in MERS-CoV, hCoV-OC43, and hCoV-HKU1 [24,29]. This PRRA site has been found to be necessary for efficient spike cleavage, cell entry, cell-to-cell fusion, and overall viral replication and pathogenesis of SARS-CoV-2 with its deletion attenuating replication and disease [30]. The insertion of a furin cleavage site at the S1/S2 cleavage site may contribute to the wider infection range of SARS-CoV-2 compared to SARS-CoV by expanding the pool of proteases priming entry. Similarly, influenza viruses with a multibasic cleavage sites, mainly those in the H5 and H7 subtypes, have increased virus pathogenicity compared to the H1, H2, H3, and H9 subtypes with monobasic sites [31].

### Making itself at home

**Fig 2:**
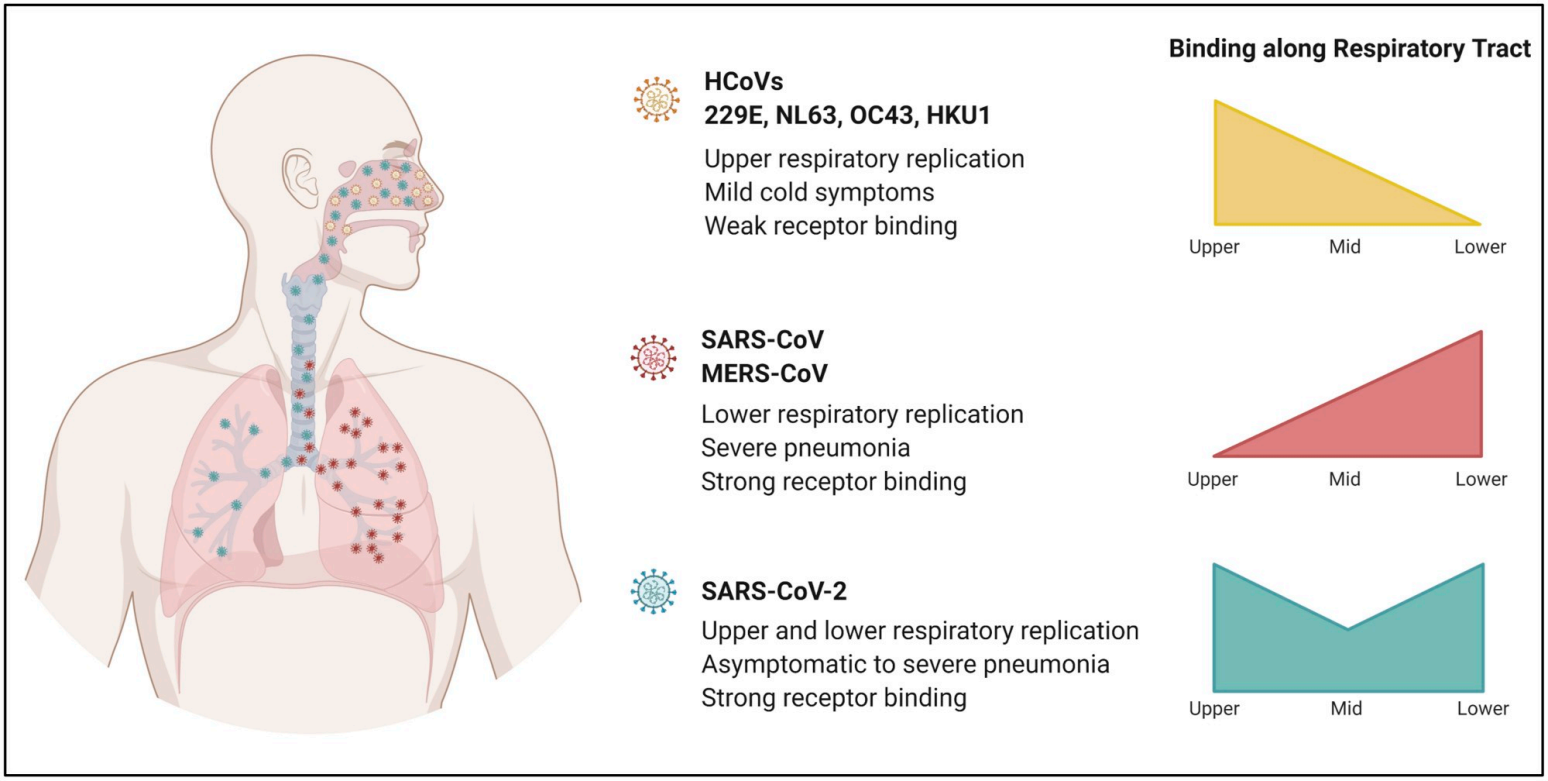
Comparison of CoV tropism. Less pathogenic hCoVs replicate in the upper respiratory tract, while highly pathogenic SARS-CoV and MERS-CoV are found to replicate in the lower respiratory tract. Viral replication is not typically observed in the mid airways for both groups. Although concentrated in the upper and lower airways, viral replication of SARS-CoV-2 has been detected throughout the respiratory tract. The ability of SARS-CoV-2 to infect and replicate throughout the airways allows for greater range in transmissibility and pathogenicity. The figure was generated using BioRender software. CoV, coronavirus; hCoV, human coronavirus; MERS-CoV, Middle East Respiratory Syndrome Coronavirus; SARS-CoV, Severe Acute Respiratory Syndrome Coronavirus; SARS-CoV-2, Severe Acute Respiratory Syndrome Coronavirus 2.

Receptor affinity and protease usage are crucial in the determining tropism (Fig 2). Any change affecting those factors would alter how well a virus transmits and its pathogenicity. Importantly, SARS-CoV-2 is making itself more at home in the host as mutations impacting both receptor binding and proteolytic cleavage have been observed in SARS-CoV-2 variants [32]. While research focus has centered on changes in the receptor-binding domain (RBD), variant mutations outside the RBD have been shown to be important. For example, the D614G variant quickly overtook the original SARS-CoV-2 strain [33–36]; positioned in the carboxyl-terminal domain of the spike protein but outside the RBD, D614G influences receptor interaction through the orientation of the spike in the up conformation [34]. Both pseudotyped virus and engineered infectious SARS-CoV-2 harboring D614G studies have shown that the D614G mutation increases virus infectivity, which, in turn, would lead to increased transmission [34–37]. The D614G mutation coupled with other RBD changes show that variant mutations impact receptor binding and affinity.

The impact of variant mutations is not limited to receptor binding and affinity. Two variants of concerns, alpha (B.1.1.7) and delta (B.1.617), have mutations within the furin cleavage site (P681H and P681R) found in the spike protein of SARS-CoV-2. Both SARS-CoV-2 variants have been observed to have increased transmission based on human cases [38,39]. In addition, our groups has demonstrated that B.1.1.7 has a replication and transmission advantage in human airway cells and the hamster models of infection, respectively [40]. Importantly, P681H and P681R in the furin cleavage site of the variant spike proteins may contribute to these differences. Initial findings with the delta variant show increased processing of the spike protein as compared to the original wild-type (WT) SARS-CoV-2 [41]. Converse to studies that removed the furin cleavage site [30], the presence of P681R mutations shifts processing more clearly to the S1/S2 cleavage product. This may contribute to changes in transmission and pathogenicity. The results also suggest an importance for proteolytic activity in SARS-CoV-2 pathogenesis and transmission.

## Conclusions

The emergence and continued circulation of SARS-CoV-2 highlight the ongoing threat posed by emerging respiratory viruses. The location, receptor binding, and proteolytic activation of CoVs dictates critical elements of transmissibility and pathogenicity. In this regard, SARS-CoV-2 has achieved an unfortunate balance, allowing for a worldwide outbreak with parallels to the 1918 H1N1 outbreak from a century earlier. Importantly, understanding how replication location, receptor interactions, and protease activation drive virus emergence and spread is paramount in disrupting and possibly preventing future emergence events.
